# Maternal repressed anger and child behavioral problems in cerebral palsy: a Bayesian path analysis

**DOI:** 10.3389/fpsyt.2025.1637492

**Published:** 2026-01-12

**Authors:** Pınar Algedik, Duygu Kurtuluş, Ayşe Merve Ata, Gülhan Sözen, Ezgi Şen Yılmaz, Bahadır Turan

**Affiliations:** 1 Department of Psychiatry, Haliç University, Istanbul, Türkiye; 2 Department of Physical Therapy and Rehabilitation, Ümraniye Education and Research Hospital, Istanbul, Türkiye; 3 Department of Physical Therapy and Rehabilitation, Ankara Bilkent City Hospital, Ankara, Türkiye; 4 Department of Pediatrics, Bahçeşehir University Faculty of Medicine, Istanbul, Türkiye; 5 Department of Psychology, Rumeli University, Istanbul, Türkiye; 6 Department of Child and Adolescent Psychiatry, Karadeniz Technical University, Trabzon, Türkiye

**Keywords:** cerebral palsy, maternal repressed anger, maternal mental health, child behavioral problems, parenting attitudes, Bayesian path analysis, psychopathology transference, caregiver burden

## Abstract

Cerebral palsy (CP) is a neurodevelopmental disorder frequently accompanied by psychiatric comorbidities, and caring for children with CP imposes a significant psychological burden on mothers. However, studies examining the relationship between maternal repressed anger and children’s behavioral problems, particularly the underlying psychopathological transference mechanisms, remain limited. This cross-sectional, case-control study aimed to investigate the associations among repressed anger, depression, anxiety, and stress in mothers of children with CP and their relationship with children’s behavioral problems using Bayesian path analysis. The study included 28 children with CP and their mothers as the patient group and 60 healthy children and their mothers, matched sociodemographically, as the control group. Children were evaluated using the Child Behavior Checklist for Ages 4–18, and mothers were assessed with the Depression Anxiety Stress Scale-Short Form, the State-Trait Anger Scale, and the Parent Attitudes Scales for Parents of Children aged 4–12 years. Six Bayesian path models were developed exclusively for the CP group to examine the dynamics of mother–child psychological interactions across behavioral domains. Mothers of children with CP exhibited significantly higher anxiety (*p* = 0.023), stress (*p* = 0.035), trait anger (*p* = 0.003), and expressed anger (*p* = 0.001) levels than controls. Bayesian modeling revealed that repressed anger increased depression (β = 0.585–0.668) and stress (β = 0.591–0.668) in mothers, which subsequently influenced children’s internalizing (β = –0.388–0.618) and externalizing (β = 0.519–0.865) problems. The models explained 20.7%–40.4% of the variance in children’s behavioral problems. These findings indicate that maternal repressed anger indirectly contributes to child psychopathology and highlight the importance of integrating mother-focused interventions targeting anger and emotion regulation into CP rehabilitation programs.

## Highlights

- Maternal mental health problems, particularly suppressed anger, were significantly associated with internalizing and externalizing psychopathologies in children with cerebral palsy.
- Elevated maternal depression and anxiety may adversely influence children’s emotional well-being through maladaptive parenting behaviors.
- Family-centered care for children with CP should include routine maternal mental health screening.
- Interventions that target maternal distress and emotion regulation may help reduce psychopathological risks in children.
- Pediatric rehabilitation programs should integrate psychosocial support for caregivers to enhance long-term developmental outcomes.

## 1 Introduction

Cerebral palsy (CP) is a neurodevelopmental disorder characterized by movement and postural impairments resulting from non-progressive brain injury during early development (1). The prevalence of CP ranges between 1.5 and 3 per 1000 live births, although this rate has gradually declined with advances in medical care (2, 3). Beyond the motor impairments typically associated in CP, children frequently exhibit psychiatric comorbidities such as intellectual disability, attention deficit hyperactivity disorder (ADHD), anxiety, depression, and conduct problems (4–7). These comorbidities complicate treatment by impairing adherence to rehabilitation programs, reducing functional gains, and negatively affecting family functioning and overall quality of life of children with CP and their families (8).

Chronic neurodevelopmental conditions such as CP impose considerable caregiving demands on parents, leading to significant psychological stress (9). Studies have consistently reported higher levels of depression, anxiety, and burnout among parents, particularly mothers, of children with CP, compared with those of typically developing children (10–12). Persistent caregiving burden, uncertainty about prognosis, and limited social support can adversely affect maternal mental health, resulting in emotional regulation difficulties such as anger suppression (13). In psychoanalytic literature, repressed anger is described as a complex defense mechanism, particularly relevant among individuals facing chronic stress. Carveth (2021) defined it as the unconscious inhibition of unexpressed aggression, which may manifest as anxiety, depression, or psychosomatic symptoms (14). Within caregiving contexts, this suppression often coexists with ambivalent feelings of love and resentment, along with guilt associated with perceived inadequacy, highlighting its dual nature as both an intrapsychic defense and a relational process that shapes the family emotional climate (15). In mothers caring for children with CP, the interplay of guilt, cultural expectations, and caregiving fatigue may heighten emotional repression. Cultural norms that emphasize resilience, such as the belief “mothers must be strong,” may further reinforce suppression of negative emotions (16). Over time, this suppression can delay psychological adaptation and intensify symptoms such as anger, depression, and burnout. Understanding these mechanisms is essential for interpreting the emotional transmission pathways explored in this study and for informing psychologically based intervention. Although elevated stress and depression have been well documented among mothers of children with CP, the mechanisms underlying the transgenerational transmission of repressed anger remain poorly understood. Such emotional strains may influence how mothers engage with their children, potentially affecting everyday interactions and the broader parent–child relationship.

The parent-child relationship plays a pivotal role in shaping children’s developmental and emotional outcomes (17). Mothers of children with CP often serve as primary caregivers, and their close interactions provide the foundation for the child’s self-development, emotion regulation, and socioemotional adaptation (18). In the context of chronic conditions such as CP, both child- and parent-related challenges can disrupt parent-child interactions, hindering healthy development and the rehabilitation processes (19). Supportive and nurturing parenting attitudes enhance the self-efficacy and emotion regulation skills of children with CP, whereas harsh or inconsistent attitudes may impede their emotional and social adjustment (20). Doğan and Demirli (2020) reported a significant association between maternal anger levels and parenting attitudes (21). Beyond anger, maternal psychological distress, particularly depression and anxiety, has been shown to influence parenting practices, often resulting in less consistent and less supportive behaviors (22, 23). These maladaptive parenting patterns are closely linked to children’s emotional and behavioral problems, suggesting an indirect pathway through which maternal psychological well-being affects child outcomes (22). Similarly, repressed anger has been reported to compromise mothers’ physical and psychological health, potentially contributing to adverse mental health outcomes in their children (24).

Family-centered perspectives emphasize the importance of supporting parental well-being as part of child-focused care (25). However, most studies on families of children with CP have employed cross-sectional designs and have not adequately captured the dynamic nature of mother–child psychological interactions.

Further research is needed to elucidate how the psychological states of mothers of children with CP influence their parenting attitudes and children’s mental health outcomes (18, 26). Although maternal distress has been widely studied, empirical evidence on the specific role of repressed anger in this population remains scarce. This represents a significant research gap, as chronic caregiving demands, feelings of guilt, role imbalances, and limited social support can promote emotional suppression and lead to psychopathological symptoms such as depression, anxiety, and burnout (27). These burdens are often compounded by psychosocial isolation and economic difficulties. To address this gap, the present study examines a broad range of maternal psychological variables with a specific focus on repressed anger and its potential indirect effects on children’s behavioral outcomes using Bayesian path modeling. This approach provides a novel perspective for understanding the mechanisms underlying mother-child psychopathological interactions in the context of CP and highlights the need for targeted, mother-focused psychosocial interventions.

The present study investigated the interaction between the psychopathological features of children with CP and their mothers. Specifically, it aimed to: (1) compare the demographic, behavioral, and psychological characteristics of children with CP and their mothers with those of typically developing children and their mothers; (2) examine the associations between maternal psychological variables, depression, anxiety, stress, anger, and parenting attitudes, and behavioral problems in children with CP; (3) determine whether maternal educational level is related to behavioral outcomes in children with CP; and (4) model the indirect effects of maternal repressed anger on children’s behavioral outcomes through maternal psychopathological features using Bayesian path analysis conducted exclusively in the CP group.

These analyses are expected to provide a clearer understanding of psychopathological patterns specific to mothers of children with CP. By elucidating the interactional structures linking maternal psychological states with children’s psychopathological outcomes, this study seeks to establish an empirical basis for developing family-centered psychosocial intervention and facilitating the early identification of at-risk groups (12, 19, 20).

## 2 Materials and methods

### 2.1 Study design and setting

This cross-sectional, case-control study was conducted between January 2025 and May 2025 at a pediatric rehabilitation clinic that provides neurodevelopmental treatment and family support programs. The study protocol was reviewed and approved by the Non-Interventional Clinical Research Ethics Committee of Haliç University (Decision No: 240, Date: 27.12.2024). All study procedures adhered to the ethical principles outlined in the Declaration of Helsinki (1964) and its subsequent amendments (28). Written informed consent was obtained from all participating mothers and, where applicable, assent was obtained from their children prior to inclusion in the study (28).

This study was not part of a larger research project. Although comprehensive sociodemographic and psychological data were collected to ensure diagnostic rigor and to characterize the sample in detail, only the variables relevant to the predefined research objectives and Bayesian path models were included in the analyses.

### 2.2 Population and sample

Children with a definitive diagnosis of CP based on the Surveillance of Cerebral Palsy in Europe (SCPE) criteria and aged 4–12 years were included in the CP group. Eligible children had a Gross Motor Function Classification System (GMFCS) level between I and V and had been residing with the same mother for at least six months. Cognitive assessments were required, and only those scoring ≥70 on the Stanford-Binet (ages 4–6) or the WISC-R (ages 6+) were included (29). Children in the control group were required to have typical development, no history of chronic, neurological, psychiatric, or neurodevelopmental disorders, and were matched to the CP group by age and sex. Mothers were eligible if they were the primary caregivers, had at least an elementary school education, and were able to independently complete the psychiatric assessment tools. Psychiatric evaluations (mothers: SCID-5-CV; children: semi-structured interviews) were administered to confirm eligibility.

Children were excluded if they had any major neurological disorder other than CP, had used psychotropic medication within the previous six months, had an acute medical condition during the study period, had severe visual or hearing impairment, or had an IQ below 70. Mothers were provided, and families received only brief feedback regarding assessment results. The control group was intentionally designed to be approximately twice the size of the CP group to enhance statistical power and ensure stability of variance estimates. Group matching was performedexcluded if they met criteria for an active psychiatric disorder according to DSM-5, had used psychotropic medication within the previous six months, or were illiterate (20). All maternal diagnoses were confirmed through structured clinical interviews conducted by licensed psychiatrists, ensuring diagnostic rigor beyond self-report measures. Although mothers completed the psychometric tools, children were evaluated directly during semi-structured interviews. Previous research indicates that behavioral assessments in children with intellectual disability below this threshold may have reduced validity; therefore, IQ < 70 was an exclusion criterion. As this study employed consecutive sampling from a single institution, the exact number of excluded cases could not be consistently recorded; however, the inclusion and exclusion process is summarized in **Supplementary Figure S1**.

### 2.3 CP diagnosis and classification criteria

CP was diagnosed by experienced pediatric neurologists in accordance with the SCPE criteria (30). The diagnoses were based on the presence of permanent but variable disorders of posture and movement resulting from nonprogressive brain lesions or anomalies occurring during early neurodevelopment. Cranial magnetic resonance imaging (MRI) findings were reviewed for all participants to confirm the diagnosis.

Based on the dominant motor pattern, children were categorized into subtypes, spastic (unilateral or bilateral), dyskinetic (dystonic or choreoatetoic), or ataxic, according to the current SCPE neurological classification system. Classification was determined by consensus between a pediatric neurologist and a physiatrist (31).

Motor function was evaluated using two standardized systems: the Gross Motor Function Classification System (GMFCS) and the Manual Ability Classification System (MACS). The GMFCS, administered by a physical medicine and rehabilitation specialist, classifies motor ability across five levels ranging from independent walking (Level I) to complete dependence (Level V) (29). The MACS assesses the child’s ability to handle objects in daily life, also on a five-level scale (32).

### 2.3 Data collection tools

#### 2.3.1 Sociodemographic data form

A comprehensive sociodemographic data form was administered for each participant. The form collected detailed information on the child’s age, sex, developmental history (including prematurity, hypoxia, and neonatal jaundice), and accompanying health problems (such as vision, hearing, and speech disorders). Functional status was assessed using the GMFCS and MACS scores. Additional variables included CP subtypes (spastic, dyskinetic, or ataxic), educational status of the child, and maternal characteristics such as age, educational attainment, and employment status. Lifestyle and socioeconomic indicators, including maternal exercise habits, family monthly income (categorized as low, medium, or high), and the number of other children in the household, were also recorded.

#### 2.3.2 Child Behavior Checklist for Ages 4–18

The CBCL/4-18 was developed by Achenbach and Edelbrock, is used to evaluate behavioral and emotional problems in children and adolescents aged 4–18 years (33). The checklist comprises 113 items rated on a 3-point Likert scale (0 = not true, 1 = somewhat or sometimes true, 2 = very true or often true). It includes eight subscales: Anxiety/Depression, Social Withdrawal, and Somatic Complaints, grouped as Internalizing Problems, and Delinquent Behaviors and Aggressive Behaviors, grouped as Externalizing Problems, along with Social Problems, Thought Problems, and Attention Problems. The validity and reliability of the Turkish version of the CBCL/4–18 were established by Erol et al. (34).

#### 2.3.3 Depression Anxiety Stress Scale – Short Form

The DASS-21 is the shortened version of the original 42-item DASS developed by Lovibond and Lovibond (35). It comprises 21 items divided into three subscales, with Depression, Anxiety, and Stress, with seven items per subscale. Each item is rated on a 4-point Likert scale reflecting the severity or frequency of symptoms experienced over the previous week. The validity and reliability of the Turkish version of the DASS-21 were established by Sarıçam (36), who reported internal consistency coefficients of 0.89, 0.85, and 0.84 for the Depression, Anxiety, and Stress subscales, respectively.

#### 2.3.4 State-Trait Anger Scale

The STAS, developed by Spielberger, is designed to assess anger intensity and patterns of anger expression (13). The STAS comprises two primary dimensions: Trait Anger (10 items) and Anger Expression (24 items). The Anger Expression domain includes three subscales: Anger-In (8 items), Anger-Out (8 items), and Anger Control (8 items). Each item is rated on a 4-point Likert scale ranging from 1 (almost never) to 4 (almost always), reflecting the frequency or typical expression of anger-related responses. The validity and reliability of the Turkish version of the STAS were established by Öner and LeCompte (37). The internal consistency coefficients were 0.82, 0.78, 0.84, and 0.73 for the Trait Anger, Anger-In, Anger-Out, and Anger Control subscales, respectively, while the test-retest reliability coefficients ranged from 0.76 to 0.88.

#### 2.3.5 Parents Attitudes Scales for parents of children aged 4 to 12 years

The PAS, developed by Gülay Ogelman and Özyürek, consists of 27 items divided into two subscales: the Desired Parental Attitude subscale (14 items) and the Undesired Parental Attitude subscale (13 items) (38). Each item is rated on a 5-point Likert scale ranging from 1 (never) and 5 (always). The Desired Parents Attitude subscale reflects positive and supportive parenting behaviors, including democratic communication, emotional warmth, and encouragement of autonomy. In contrast, the Undesired Parental Attitude subscale reflects negative or maladaptive parenting behaviors, such as authoritarian control, rejection, overprotection, or inconsistent discipline. Higher scores on the Desired Attitude subscale indicate more adaptive parenting attitudes, whereas higher scores on the Undesired Attitude subscale indicate less adaptive parenting attitudes. The internal consistency (Cronbach’s alpha) coefficients were reported as 0.81 for the Desired Attitude subscale and 0.748 for the Undesired Attitude subscale, demonstrating satisfactory reliability.

### 2.5 Data collection procedure

Families of children with CP were consecutively recruited from the pediatric rehabilitation and neurodevelopmental clinic of Haliç University, while the control group was recruited from healthy children attending the general pediatric outpatient clinic of the same institution. No financial or material incentives were provided, and families received only brief feedback regarding assessment results. The control group was intentionally designed to be approximately twice the size of the CP group to enhance statistical power and ensure stability of variance estimates. Group matching was performed based on the age and sex of the children, whereas other sociodemographic differences reflected the natural distribution of the CP population.

Data collection was conducted in three stages. In the first stage (approximately 20 minutes), semi-structured interviews were conducted with both the mother and the child to establish rapport and gather initial background information. In the second stage (30–40 minutes), the child underwent a comprehensive psychiatric assessment by a child psychiatrist, while the mother simultaneously completed the relevant psychometric instruments (CBCL/4–18, DASS-21, STAS, and PAS) under the supervision of a clinical psychologist. Children did not complete any self-report measures; instead, they were directly evaluated through clinician-administered, semi-structured interviews. This design ensured that maternal reports and the clinician-based child assessments provided complementary perspectives. In the final stage (20–30 minutes), mother-child interactions were observed in a naturalistic setting to evaluate behavioral and emotional exchanges.

All assessments were scheduled between 9.00 am and 12.00 pm to minimize fatigue and medication-related effects. During sessions, a physiotherapist assisted in ensuring the children’s comfort and mobility. Considering the motor and communicative limitations associated with CP, additional time and rest breaks were provided when necessary. Mothers’ comprehension of questionnaire items was verified throughout the process, with clarifications provided when needed to maintain accuracy and reliability. Age-appropriate reinforcers were used to sustain the children’s motivation during evaluations. At the end of each assessment session, brief feedback was provided to both the mother and child, and additional recommendations for further assessment or intervention were offered when indicated.

### 2.6 Statistical analyses

A Bayesian estimation method was preferred over classical maximum likelihood (ML) estimation, as it provides more robust parameter estimates in studies with moderate sample sizes, incorporates prior information, and yields posterior distributions with credibility intervals that better reflect parameter uncertainty (39). The sample size was determined according to the fundamental principles and evaluation criteria of Bayesian modeling rather than through a traditional power analysis used in frequentist approaches (40). In Bayesian estimation, model adequacy is not evaluated through type I and type II error rates but rather by examining the model’s fit to the data, posterior parameter distributions, predictive power, and variance explanation rates (40). Accordingly, all structural models developed in this study were evaluated using posterior predictive probability (PPP) values, posterior standard residual values, 95% credibility intervals (CrIs) of the path coefficients, and R² values, and all estimated path coefficients were found to be statistically significant, as their 95% CrIs did not include zero.

Based on posterior fit indicators, including PPP and posterior standard residual values, the CrIs of path coefficients, and R² explanatory values, applied as an alternative to traditional power analysis, the sample size was considered sufficient to ensure model reliability. These metrics collectively demonstrated that the models provided consistent, theoretically coherent, and statistically robust results.

For clarity and consistency with the Results section, analyses were conducted in the following sequence: descriptive statistics, group comparisons, correlation analyses, and Bayesian path modeling. Descriptive statistics were presented as mean ± standard deviation for continuous variables following a normal distribution or as median (minimum–maximum) for non-normally distributed variables. Categorical variables were summarized as counts (n) and percentages (%). The normality of continuous variables was assessed using the Shapiro–Wilk test. In addition to significance testing, effect sizes were calculated (Cohen’s d for continuous variables and Cramer’s V for categorical variables) to facilitate interpretation, given the relatively small sample size of the CP group (41, 42). For Bayesian path models, standardized path coefficients and their 95% CrIs were reported as indicators of effect size. Between-group comparisons were performed using the independent samples *t*-test for normally distributed variables and the Mann–Whitney *U*-test for non-normal variables. Differences in categorical variables were evaluated using Pearson’s chi-square test for 2x2 tables with expected cell counts ≥5, Fisher’s exact test for 2x2 tables with expected cell counts <5, and Fisher–Freeman–Halton test for RxC tables.

The relationships between the behavioral problems of children with CP and the psychological states of their mothers were examined using Spearman’s correlation analysis. The association between children’s behavioral problems and maternal educational status were evaluated using the Kruskal-Wallis test. Maternal education was analyzed as a key sociodemographic variable because it is a well-established indicator of parenting practices and child developmental outcomes (43, 44). Although employment status is also an important socioeconomic factor, it was excluded from subgroup comparisons due to its greater heterogeneity and instability within the sample, which limited its interpretability. Therefore, maternal education was selected as the primary variable for subgroup analyses.

Path models based on the Bayesian estimation method were employed to examine the effects of maternal emotional characteristics on psychological outcomes. These analyses were conducted exclusively within the CP group, as the primary objective was to model the indirect effects of maternal repressed anger on the behavioral outcomes of children with CP. Separate path models were developed for each psychological variable representing child outcomes, and the fit of each model was evaluated.

Successive model updates were performed to determine the structure that best explained the observed relationships among variables. Model performance was assessed using standard fit criteria, including PPP, posterior standard residual, and Akaike Information Criterion (AIC) values. The model demonstrating the optimal theoretical coherence and statistical fit was selected as the final version.

The Bayesian estimation method was used to analyze the path models. This probabilistic approach updates parameter estimates based on prior information, generating posterior distributions that reflect parameter uncertainty. Model parameters were estimated using the Metropolis–Hastings (MH) algorithm, a Markov chain Monte Carlo (MCMC) sampling technique that iteratively draws samples from successive probability distributions (39).

To ensure convergence, 10000 iterations were performed for each model, and the Potential Scale Reduction (PSR) was computed using at least two independent chains (45). A thinning value of 10 was applied to enhance sampling efficiency and reduce autocorrelation among successive samples. Point estimates were calculated using the posterior mean method.

The direct effects and covariance relationships among the variables were evaluated within a Bayesian estimation framework. Indirect effects in the path models were calculated by multiplying the posterior distributions of the relevant path coefficients, and their 95% CrIs were derived from the posterior draws (40). For all mean parameters, mean coefficient values (B), standard errors (SE), 95% CrI, and PPP values were reported.

Statistical analyses were performed using Jamovi (version 2.3.28.0; The Jamovi Project, 2023; https://www.jamovi.org), JASP (version 0.19.2; Jeffreys’ Amazing Statistics Program, 2024; https://jasp-stats.org), and Mplus (version 8.3; Muthén & Muthén, 2019; https://www.statmodel.com). A two-tailed probability (*p*) value ≤ 0.05 was considered statistically significant.

## 3 Results

Before presenting the findings, it is important to briefly recall the study objectives. This section reports the results of (1) group comparisons between the CP and control groups, (2) associations between maternal psychological variables and child behavioral outcomes, and (3) Bayesian path models examining the indirect effects of maternal repressed anger. There were no significant differences in age or sex between the patient and control groups (*p* > 0.05). However, the proportion of participants with abnormal findings in their medical history was significantly higher in the patient group than in the control group (78.6% vs. 21.7%, *p* < 0.001). In particular, the rate of premature birth was markedly higher in the patient group compared to the control group (63.6% vs. 7.7%, *p* < 0.001). Similarly, additional health problems were significantly more common in the patient group (53.6% vs. 5.0%, *p* < 0.001). Among these, comprehension and vision impairments were the most frequent, each observed in 33.3% of patients. Moreover, the majority of children with CP (63.0%) received special education, whereas most children in the control group (96.4%) attended mainstream schools (*p* < 0.001).

A comparison of the mothers of children in the patient and control groups revealed that the mean age of mothers of children with CP was significantly lower than that of mothers in the control group (33.5 years vs. 38.0 years, *p* < 0.001). None of the mothers of children in the CP group were employed, and their educational level was significantly lower than that of mothers in the control group (*p* < 0.001). The proportion of children with siblings was significantly higher in the CP group than in the control group (92.9% vs. 36.7%; *p* < 0.001), and the median number of siblings was also higher in the CP group (2.0 vs. 1.0, *p* = 0.003). Effect sizes for continuous variables were as follows: Age (Child), d = 0.12; Mother’s age, d = 0.73; Number of siblings, d = 0.95. For categorical variables, the corresponding Cramer’s V values were: Gender, V = 0.01; Medical history, V = 0.54; Additional problems, V = 0.56; Education (present), V = 0.06; Education type, V = 0.66; Mother’s employment status, V = 0.30; Mother’s educational level, V = 0.92; Siblings (present), V = 0.53 (**Table 1**).

**Table 1:** Demographic and clinical characteristics of children with cerebral palsy and control group.

Variable		Patient(n = 28)	Control(n = 60)	*p*	Effect size
Age (Child)		6.0 [4.0–15.0]	6.0 [3.0–17.0]	0.577	d=0,124
Gender (Child)	Female	13 (46.4)	27 (45.0)	0.999	v=0.013
	Male	15 (53.6)	33 (55.0)		
Medical History (Child), Present		22 (78.6)	13 (21.7)	**<0.001**	v=0.542
Medical History (Child)	Prematurity	14 (63.6)	1 (7.7)	**<0.001**	
	Incubator Care	6 (27.3)	3 (23.1)		
	Neonatal Jaundice	2 (9.1)	9 (69.2)		
Additional Problems, Present		15 (53.6)	3 (5.0)	**<0.001**	v=0.561
Additional Problems	Seizures	3 (20.0)	–	–	
	Comprehension	5 (33.3)	–		
	Vision	5 (33.3)	–		
	Speech	0 (0.0)	–		
	Swallowing	2 (13.3)	–		
Functional Status	Mobilization with Support	13 (46.4)	–	–	
	Mobile	7 (25.0)	–		
	Immobile	5 (17.9)	–		
	Normal	3 (10.7)	–		
FAS	0	5 (17.9)	–	–	
	1	2 (7.1)	–		
	2	12 (42.9)	–		
	4	3 (10.7)	–		
	5	6 (21.4)	–		
GMFCS	1	7 (25.0)	–	–	
	2	7 (25.0)	–		
	3	9 (32.1)	–		
	4	0 (0.0)	–		
	5	5 (17.9)	–		
CP Type	Quadriparetic	8 (28.6)	–	–	
	Diplegic	17 (60.7)	–		
	Hemiplegic	3 (10.7)	–		
Education, Present		27 (96.4)	56 (93.3)	0.999	v=0.062
Education	School	10 (37.0)	54 (96.4)	**<0.001**	v=0.662
	Special Education	17 (63.0)	2 (3.6)		
Mother’s Age		33.5 [26.0 – 55.0]	38.0 [26.0 – 50.0]	**<0.001**	d=0,725
Mother’s Employment Status	Yes	0 (0.0)	14 (23.3)	**0.004**	v=0.297
	No	28 (100.0)	46 (76.7)		
Mother’s Educational Status	Elementary School	10 (35.7)	0 (0.0)	**<0.001**	v=0.922
	Middle School	8 (28.6)	0 (0.0)		
	High School	7 (25.0)	0 (0.0)		
	University	3 (10.7)	59 (98.3)		
	Graduate School	0 (0.0)	1 (1.7)		
Siblings, Present		26 (92.9)	22 (36.7)	**<0.001**	v=0.526
Number of Siblings		2.0 [1.0–4.0]	1.0 [1.0–3.0]	**0.003**	d=-0,954

**Bold** p-values indicate statistical significance (p ≤ 0.05).

Descriptive statistics are presented as n (%) for categorical variables and as medians [minimum] for numerical variables. Effect sizes are reported as Cohen’s d for continuous variables and Cramer’s V for categorical variables. CP, Cerebral Palsy; GMFCS: Gross Motor Function Classification System; FAS: Functional Ambulation Scale.

Comparison of the patient and control groups in terms of behavioral and psychological characteristics showed that the median CBCL/4-18 “Somatic Complaints” subscale score was significantly higher in the CP group than in the control group (*p* = 0.022), whereas no significant differences were found between the groups for the other seven CBCL/4–18 subscales (**Table 2**). The median DASS-21 anxiety and stress subscale scores of mothers of children with CP were also significantly higher than those of mothers in the control group (*p* = 0.023 and *p* = 0.035, respectively). Furthermore, the prevalence of stress positivity was significantly greater among mothers of children with CP than among those in the control group (*p* = 0.012).

**Table 2:** Behavioral and psychological assessment results of children with cerebral palsy and a control group.

Variable		Patient (n = 28)	Control (n = 60)	*p*	Effect size
Child Behavior Checklist for Ages 4–18 (CBCL/4–18) ▼					
Anxiety/Depression		2.0 [0.0–17.0]	3.0 [0.0–13.0]	0.529	d = 0.076
Social Withdrawal/Depression		0.0 [0.0–10.0]	1.0 [0.0–6.0]	0.985	d = 0.142
Somatic Complaints		2.0 [0.0–10.0]	1.0 [0.0–9.0]	**0.022**	d = 0.713
Social Problems		1.0 [0.0–14.0]	2.0 [0.0–9.0]	0.834	d = 0.379
Thought Problems		1.0 [0.0–8.0]	1.0 [0.0–7.0]	0.576	d = 0.095
Attention Problems		2.0 [0.0–9.0]	2.5 [0.0–7.0]	0.744	d = 0.067
Delinquent Behaviors		1.0 [0.0–10.0]	2.0 [0.0–9.0]	0.265	d = 0.055
Aggressive Behaviors		2.0 [0.0–15.0]	3.0 [0.0–13.0]	0.071	d = 0.325
Depression Anxiety Stress Scale (DASS-21) ▼					
Depression		5.5 [0.0–19.0]	4.0 [0.0–19.0]	0.181	d = 0.365
Anxiety		4.5 [0.0–21.0]	2.0 [0.0–18.0]	**0.023**	d = 0.577
Stress		6.5 [0.0–19.0]	4.5 [0.0–21.0]	**0.035**	d = 0.476
**Depression**	Negative	13 (46.4)	36 (60.0)	0.335	V = 0.16
	Positive	15 (53.6)	24 (40.0)		
**Anxiety**	Negative	13 (46.4)	42 (70.0)	0.059	V = 0.20
	Positive	15 (53.6)	18 (30.0)		
**Stress**	Negative	15 (53.6)	49 (81.7)	**0.012**	V = 0.28
	Positive	13 (46.4)	11 (18.3)		
State-Trait Anger Scale (STAS) ▼					
Trait Anger		20.5 [12.0–40.0]	16.0 [9.0–32.0]	**0.003**	d = 0.751
Anger Control		26.0 [14.0–32.0]	24.0 [15.0–32.0]	0.185	d = 0.312
Anger-Out		16.5 [9.0–26.0]	13.0 [9.0–23.0]	**0.001**	d = 0.997
Anger-In		15.6 ± 3.8	14.9 ± 3.3	0.440	d = 0.188
Parents Attitudes Scales for Parents of Children Aged 4 to 12 Years (PAS) ▼					
Desired Attitudes		35.5 [19.0–70.0]	63.0 [50.0–70.0]	**<0.001**	d = 2.229
Unwanted Attitudes		40.0 [26.0–55.0]	25.0 [15.0–38.0]	**<0.001**	d = 2.842

**Bold** p-values indicate statistical significance (p ≤ 0.05).

Note: Descriptive statistics are presented as mean ± standard deviation or median [minimum] for numerical variables. Effect sizes are reported as Cohen’s d for continuous variables and Cramer’s V for categorical variables. CBCL: Child Behavior Checklist; DASS: Depression Anxiety Stress Scale; STAS: State-Trait Anger Scale; PAS: Parent Attitudes Scale.

Similarly, mothers of children with CP had significantly higher median STAS trait Anger and Anger-Out subscale scores than mothers in the control group (*p* = 0.003 and *p* = 0.001, respectively). Regarding parental attitudes, the median undesired PAS subscale score was significantly higher, whereas the desired PAS subscale score was significantly lower among mothers of children with CP compared with controls (*p* < 0.001 for both cases). Effect sizes for continuous variables were as follows: Somatic complaints, d = 0.71; DASS depression, d = 0.37; DASS anxiety, d = 0.58; DASS stress, d = 0.48; STAS trait anger, d = 0.75; STAS anger control, d = 0.31; STAS anger-out, d = 1.00; STAS anger-in, d = 0.19; Desired parental attitudes, d = 2.23; Unwanted parental attitudes, d = 2.84. For categorical DASS cut-off variables, the corresponding Cramer’s V values were: Depression positivity, V = 0.16; Anxiety positivity, V = 0.20; Stress positivity, V = 0.28 (**Table 2**).

Analysis of the relationships between the behavioral problems of children with CP and maternal psychological variables revealed that the children’s behavioral problems were most strongly correlated with maternal stress levels (**Table 3**). Accordingly, maternal stress showed significant positive correlation with children’s anxiety and depression (r = 0.419, *p* = 0.027), social withdrawal (r = 0.412, *p* = 0.029), social problems (r = 0.400, *p* = 0.035), thought problems (r = 0.442, *p* = 0.018), attention problems (r = 0.377, *p* = 0.048) and aggressive behaviors (r = 0.498, *p* = 0.007) subscale scores. Similarly, maternal anxiety was significantly associated with children’s social withdrawal (r = 0.410, *p* = 0.030), social problems (r = 0.395, *p* = 0.038), and aggressive behavior (r = 0.458, *p* = 0.014) subscale scores. Maternal depression correlated significantly only with the aggressive behavior subscale scores of children with CP (r = 0.430, *p* = 0.022). Analysis of the association between behavioral problems in children with CP and maternal educational levels revealed no significant differences in the CBCL/4–18 subscale scores across maternal education categories (p > 0.05).

**Table 3:** Correlation analysis between behavioral problems of children with cerebral palsy and maternal variables.

Variable		Anxiety/Depression	Social Withdrawal/Depression	Somatic Complaints	Social Problems	Thought Problems	Attention Problems	Delinquent Behaviors	Aggressive Behaviors
Depression Anxiety Stress Scale (DASS-21) ▼									
Depression	r	0.269	0.271	0.327	0.235	0.333	0.260	0.223	0.430*
Anxiety	r	0.341	0.410*	0.340	0.395*	0.364	0.271	0.355	0.458*
Stress	r	0.419*	0.412*	0.357	0.400*	0.442*	0.377*	0.362	0.498**
State-Trait Anger Scale (STAS) ▼									
Trait Anger	r	0.268	0.311	0.310	0.335	0.303	0.302	0.231	0.294
Anger Control	r	0.091	0.079	-0.110	0.062	0.174	0.179	0.055	0.064
Anger-Out	r	0.007	0.258	0.216	0.190	-0.063	-0.021	0.060	-0.032
Anger-In	r	0.266	0.237	0.190	0.285	0.383*	0.220	0.308	0.297
**FAS**	r	-0.119	0.126	-0.123	0.005	-0.030	-0.283	0.093	0.077
**GMFCS**	r	0.085	-0.079	0.038	-0.115	-0.023	0.194	-0.147	-0.077
**Mother’s Age**	r	0.390*	0.134	0.368	0.301	0.262	0.469*	0.277	0.475*
Parents Attitudes Scales for Parents of Children Aged 4 to 12 Years (PAS) ▼									
Desired Attitudes	r	0.199	0.234	0.060	0.090	0.177	0.397*	0.290	0.254
Unwanted Attitudes	R	-0.238	0.050	0.064	-0.037	-0.299	-0.049	-0.151	-0.120
**Age (Child)**	r	0.020	0.136	0.082	0.235	0.114	0.255	0.111	0.231

*p < 0.05, **p < 0.01. Unmarked coefficients are non-significant. Correlations were calculated using Spearman’s rho.

Correlations were calculated using Spearman’s correlation coefficient (r). CBCL: Child Behavior Checklist; DASS: Depression Anxiety Stress Scale; STAS: State-Trait Anger Scale; PAS: Parent Attitudes Scales; FAS: Functional Ambulation Scale; GMFCS: Gross Motor Function Classification System.

**Figure 1:**
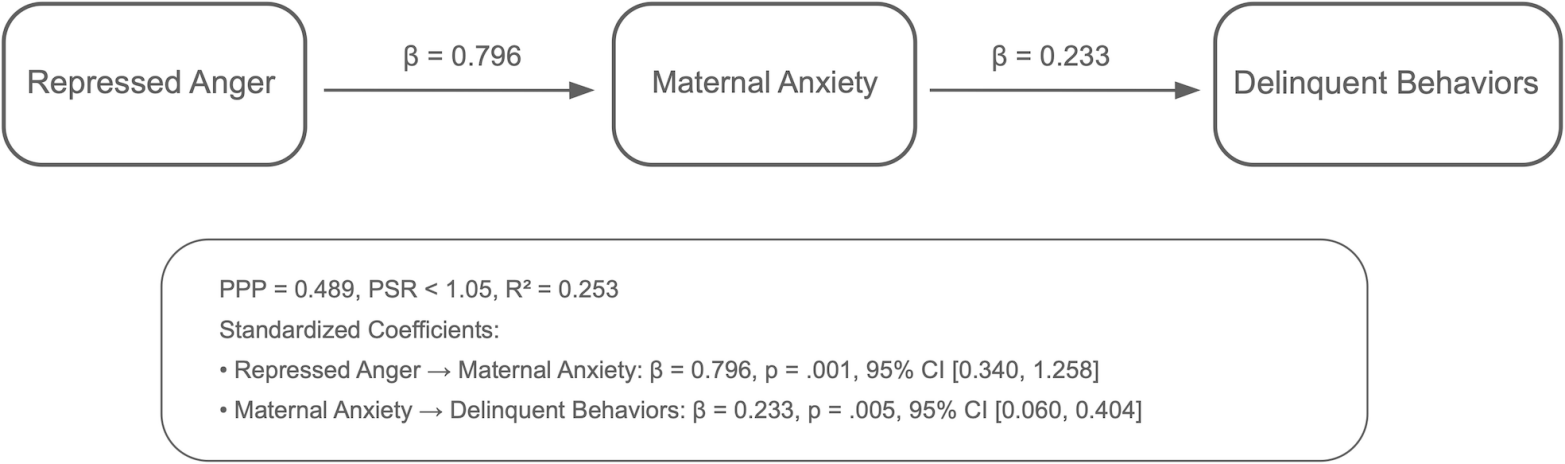
Path model showing the relationship between repressed anger, maternal depression, maternal stress, and child anxiety/depression repressed anger positively affects maternal depression and stress, while maternal depression negatively impacts child anxiety/depression symptoms, and maternal stress positively affects them. Standardized regression coefficients are shown in the figure. Model fit: posterior predictive *p* = 0.434.

**Figure 2:**
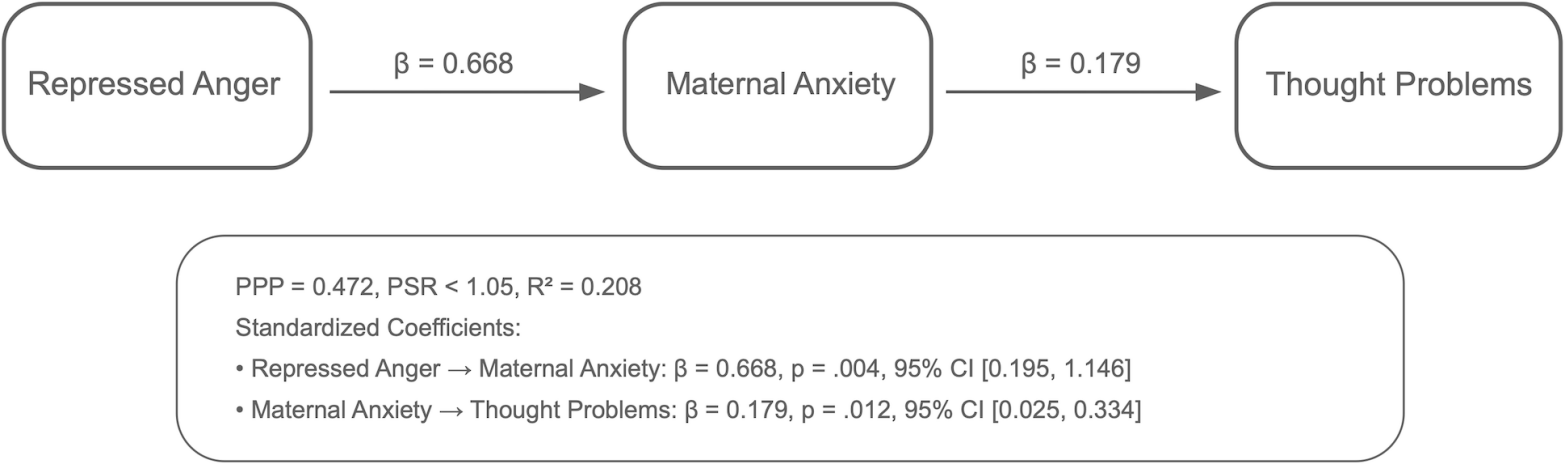
Path model showing the relationship between repressed anger, maternal stress, and attention problems in children repressed anger increases maternal stress, which in turn affects attention problems in children. Standardized regression coefficients are shown in the figure. Model fit: posterior predictive *p* = 0.331.

**Figure 3:**
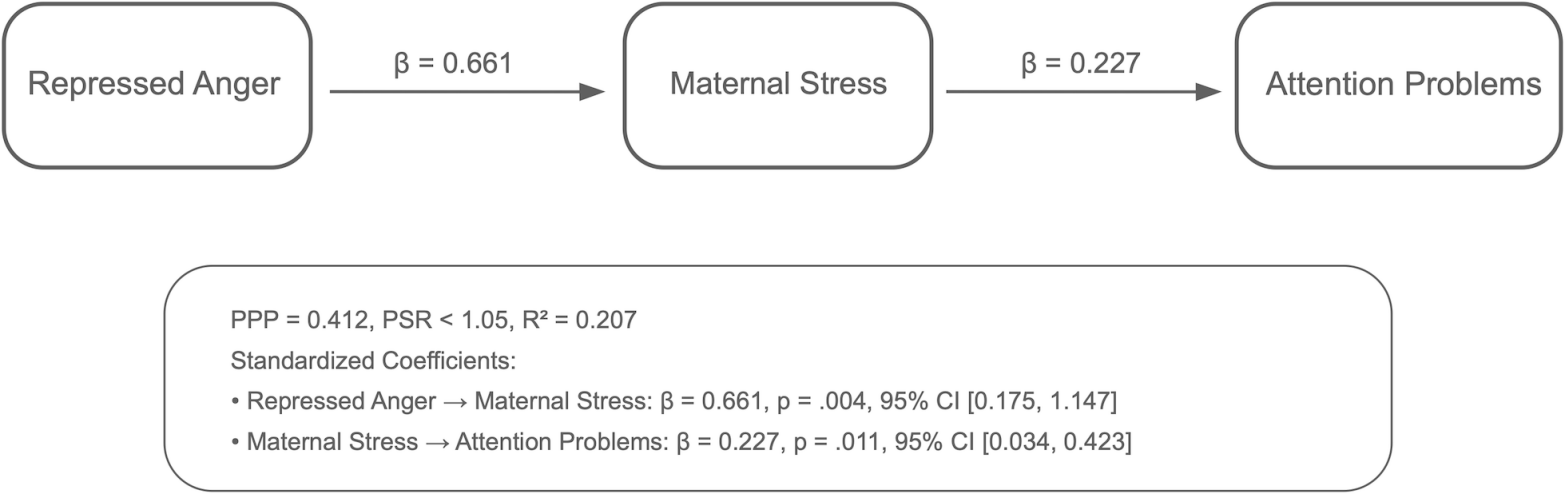
Path model showing the relationship between repressed anger, maternal anxiety, and child conduct disorder repressed anger increases maternal anxiety, which in turn affects conduct disorder in children. Standardized regression coefficients are shown in the figure. Model fit: posterior predictive *p* = 0.538.

**Figure 4:**
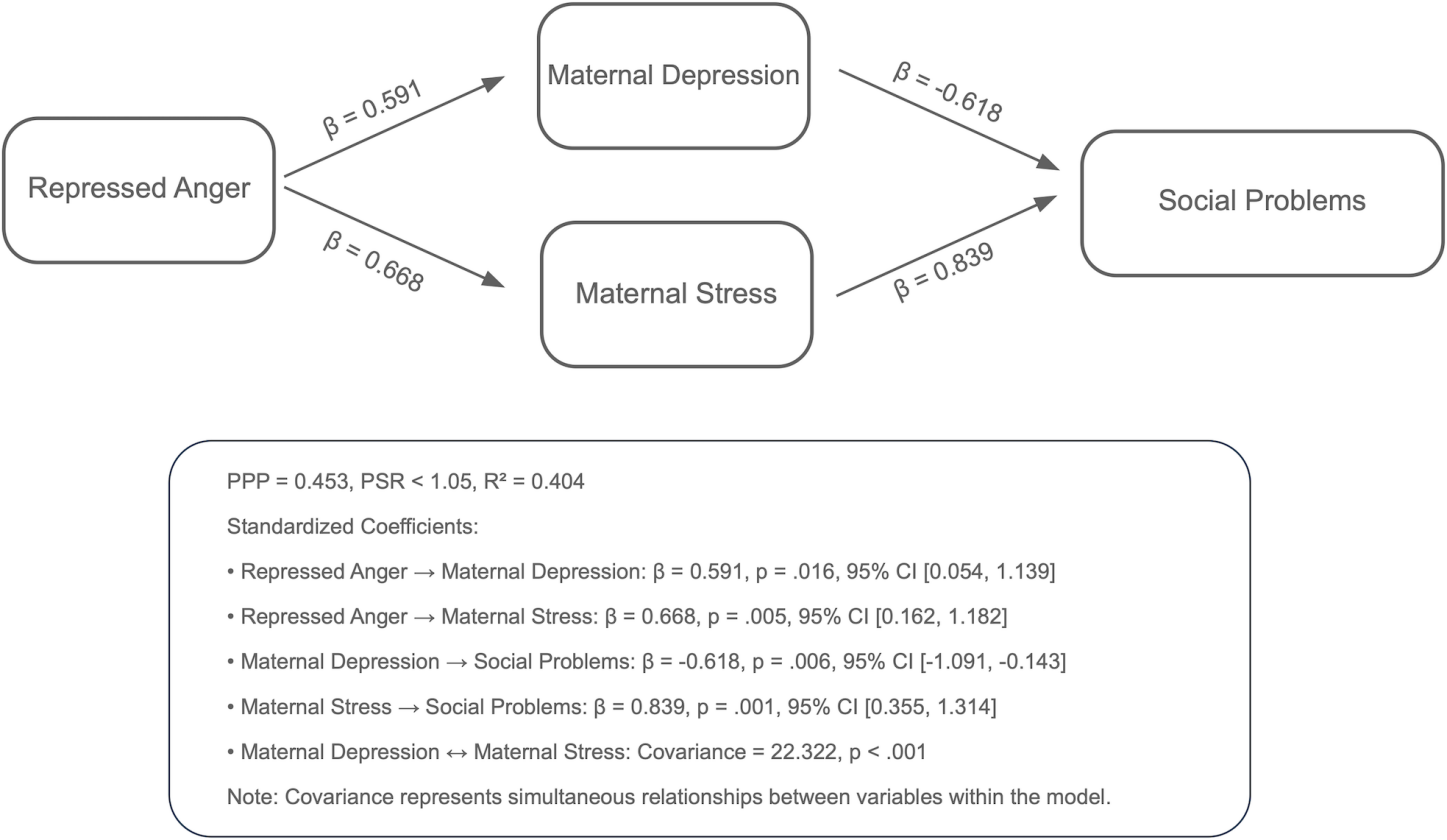
Path model showing the relationship between repressed anger, maternal depression, maternal stress, and social withdrawal/depression in children repressed anger increases maternal depression and stress, with maternal depression negatively affecting social withdrawal/depression symptoms in children and maternal stress positively affecting them. Standardized regression coefficients are shown in the figure. Model fit: posterior predictive *p* = 0.446.

**Figure 5:**
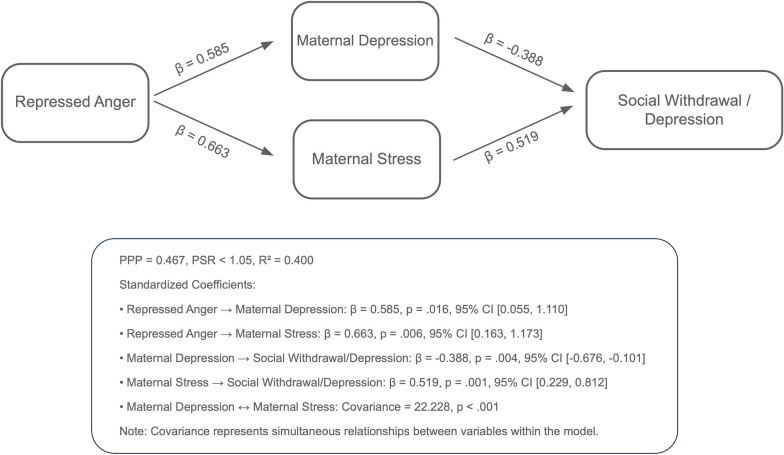
Path model showing the relationship between repressed anger, maternal depression, maternal stress, and social problems in children repressed anger increases maternal depression and stress, with maternal depression negatively affecting social problems in children and maternal stress positively affecting them. Standardized regression coefficients are shown in the figure. Model fit: posterior predictive *p* = 0.468.

**Figure 6:**
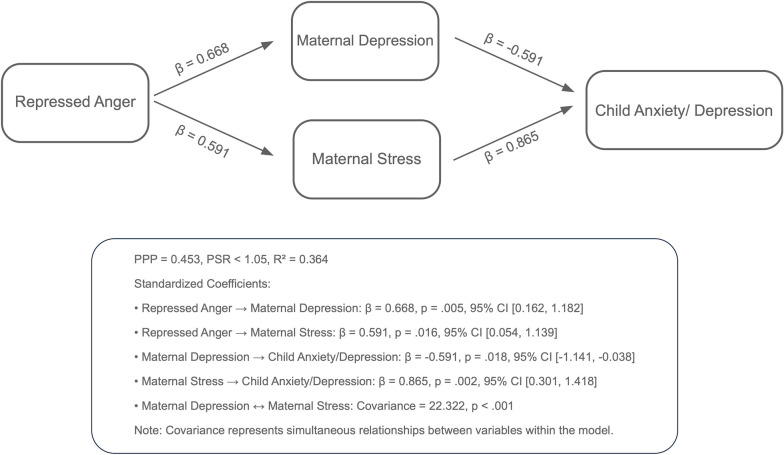
Path model showing the relationship between repressed anger, maternal anxiety, and thought problems in children repressed anger increases maternal anxiety, which in turn affects thought problems in children. Standardized regression coefficients are shown in the figure. Model fit: posterior predictive *p* = 0.536.

Analyses using the Bayesian estimation method revealed the indirect effects of repressed anger in mothers of children with CP on their children’s behavioral problems across six path models. The PPP values, the R^2^ values of the outcome variables, and standardized significant path coefficients for each model are presented in **Figures 1**–**6**. All estimated path coefficients were statistically significant, as their 95% CrIs did not include zero. All models demonstrated acceptable fit to the data, with PPP values ranging from 0.331 to 0.538, indicating that the sample size was adequate to yield reliable parameter estimates. No significant direct effects were observed from maternal repressed anger to any child behavioral outcomes; its influence was transmitted indirectly through maternal depression, anxiety, and stress.

## 4 Discussion

The key sociodemographic characteristics observed in this study indicated that mothers of children with CP had lower education levels, more children, and were all unemployed, suggesting a bidirectional relationship between CP and socioeconomic status. Low socioeconomic status may increase the risk of CP through perinatal complications, inadequate prenatal care, and limited access to healthcare services (46–48). Conversely, caring for a child with CP can further compromise a family’s socioeconomic status by restricting maternal employment and career advancements, thereby creating a self-perpetuating cycle of disadvantage. These findings are consistent with previous studies and underscore the complex interplay between CP and socioeconomic factors (10, 38).

Regarding parenting attitudes, mothers of children with CP demonstrated significantly higher scores on the undesired attitudes subscale and lower scores on the desired attitudes subscale compared with mothers of typically developing children. These findings suggest that chronic caregiving responsibilities may negatively influence parenting attitudes. Although this study did not directly examine the mechanisms linking maternal self-regulation and parenting attitudes, previous research has shown that difficulties in emotional self-regulation can adversely affect parenting practices. Davis et al. reported that the feelings of inadequacy experienced by mothers of children with CP may lead to inconsistent disciplinary behaviors (20). Similarly, Çakır and Kızıler demonstrated that chronic caregiving stress reduces parental self-efficacy, fostering more controlling or overprotective behaviors (49). Taken together, our results contribute to the existing literature by supporting evidence that caregiving demands associated with chronic childhood conditions can shape maladaptive parental attitudes and highlight the importance of supporting parental emotional regulation in this population.

Our findings revealed a robust association between maternal psychopathology and behavioral problems in children with CP. Higher maternal stress levels were significantly correlated with children’s anxiety/depression, social withdrawal, thought problems, and aggressive behaviors. Similarly, maternal anxiety and depression were positively associated with a range of child behavioral difficulties. Hermansen et al. proposed that the link between maternal depression and children’s behavioral problems may be mediated by three mechanisms: inadequate parental responsiveness, transmission of maladaptive emotion regulation patterns through social learning, and shared genetic vulnerability (50). Our findings support this framework but further suggest that these mechanisms may function differently in children with neurodevelopmental disorders such as CP. Flouri et al. further demonstrated that limitations in children’s cognitive and emotional capacities can interact with maternal psychopathology, shaping developmental outcomes (51). These findings emphasize the unique and bidirectional dynamics of mother–child interactions in CP.

The primary finding of this study, which examined the relationship between repressed anger in mothers of children with CP and their children’s behavioral problems, was that maternal repressed anger indirectly influenced children’s behavioral outcomes through psychopathological factors such as depression, anxiety, and stress. The significantly higher levels of anxiety, stress, trait anger, and expressed anger observed among mothers of children with CP, compared with mothers of typically developing children, highlight the psychological burden associated with chronic caregiving. These findings align with previous reports demonstrating elevated rates of psychopathology in parents of children with chronic illness (36, 37). A particularly important contribution of this study is the identification of a mechanism by which maternal repressed anger indirectly affects children’s behavioral functioning via increased maternal depression, anxiety, and stress. This mechanism represents one of the first systematic models to explain emotion regulation difficulties in parents of children with CP. Byrne et al. reported that suppressing negative emotions such as anger in mothers of children with CP was associated with feelings of guilt and inadequacy (13), whereas Woodgate et al. described that parents caring for children with complex medical needs often experience emotional isolation, leading to breakdowns in family communication (38). Extending these findings, our study provides quantitative evidence that repressed anger in mothers manifests as behavioral problems in their children through distinct psychopathological pathways. Integrating family-based approaches, as proposed by Dempsey et al., into CP rehabilitation programs may enhance both maternal and child psychological well-being by improving parental emotion regulation, particularly regarding anger management (52).

Building on the preceding findings, the study also revealed an unexpected negative association between maternal depression and internalizing problems in children with CP, contrary to prior evidence. This discrepancy likely reflects methodological differences. Because behavioral assessments of children with CP were based on maternal reporting, depressive symptoms may have influenced mothers’ ability to perceive and accurately evaluate their children’s emotional difficulties. Raina et al. suggested that depressed caregivers might underrecognize internalizing symptoms in their children due to preoccupation with their own emotional state (53). Clinically, these findings emphasize the need for psychiatric evaluations of children with CP to combine parental reports with clinician observations and, when possible, direct assessments of the child.

No significant effect of motor function levels, as indicated by GMFCS and MACS scores, was observed on the relationship between maternal and child psychopathology. This indicates that the diagnosis of CP, rather than its severity, has a significant psychosocial impact on family dynamics. Similarly, Raina et al. reported that a CP diagnosis can exert broad psychosocial effects on the family system, irrespective of functional impairment (53). These findings reinforce the importance of integrating family-centered support within rehabilitation programs for children with CP.

### 4.1 Limitations and strengths of the study

This study has several methodological limitations. First, although the relatively small sample size and single-center design may limit the generalizability of the findings, the robustness of the Bayesian model outcomes partially mitigates this concern. The cross-sectional design also restricted our ability to examine temporal dynamics and infer causal relationships within mother–child psychological interactions.

Although mothers with active psychiatric diagnoses were excluded based on clinical interviews and self-reports, structured diagnostic instruments (e.g., SCID-5-CV) were not employed. Consequently, the presence of subthreshold or undiagnosed mental disorders among participants cannot be ruled out, and elevated symptom scores should therefore be interpreted with caution. Future studies should incorporate structured diagnostic assessments to enhance diagnostic precision and strengthen the link between symptom severity and diagnostic threshold.

Another consideration pertains to the conceptualization and measurement of repressed anger, a latent psychodynamic construct. While the STAS provides a standardized and validated assessment of anger expression styles, the unconscious and defensive dimensions of repressed anger may not be fully captured through self-reporting instruments. This limitation may constrain the interpretive depth of the pathways connecting maternal emotional dysregulation to child behavioral outcomes.

Future research would benefit from longitudinal designs, clinician-administered diagnostic instruments, and multi-informant behavioral assessments to achieve a more comprehensive understanding of mother–child psychopathological transmission.

Despite these limitations, the study also possesses several strengths. It is among the first to quantitatively model the indirect effects of maternally repressed anger on child behavioral outcomes in a CP population using Bayesian path analysis, a robust approach suited for moderate sample sizes. The use of validated psychometric tools, systematic clinical interviews for participant screening, and the integration of maternal self-reports with clinician-based child assessments further strengthen the reliability of the findings. Collectively, these features enhance the study’s credibility and provide valuable insights to inform the development of family-centered psychosocial interventions in pediatric CP rehabilitation.

## 5 Conclusions

In conclusion, the present findings highlight the need to integrate mother-focused psychological interventions within rehabilitation programs for children with CP. Enhancing the emotion regulation abilities of mothers, particularly their capacity to manage repressed anger and stress, may improve both maternal psychological well-being and the child’s behavior outcomes. Pediatric rehabilitation teams should adopt a holistic approach that considers the family system as a unit of care and includes targeted support for maternal psychosocial needs in treatment planning. Future research should examine the long-term efficacy of such interventions and their applicability across different CP subtypes and contexts.

## Data Availability

The raw data supporting the conclusions of this article will be made available by the authors, without undue reservation.

## Supplementary material

Supplementary Figure S1 Flowchart illustrating the recruitment, screening, exclusion, and final inclusion of participants in the study. CP = Cerebral Palsy.
