# Supplementary figures and images for: Maternal repressed anger and child behavioral problems in cerebral palsy: a Bayesian path analysis
Source: Front Psychiatry. 2026 Jan 12;16:1637492. doi: 10.3389/fpsyt.2025.1637492 (PMC12833217; doi:10.3389/fpsyt.2025.1637492)

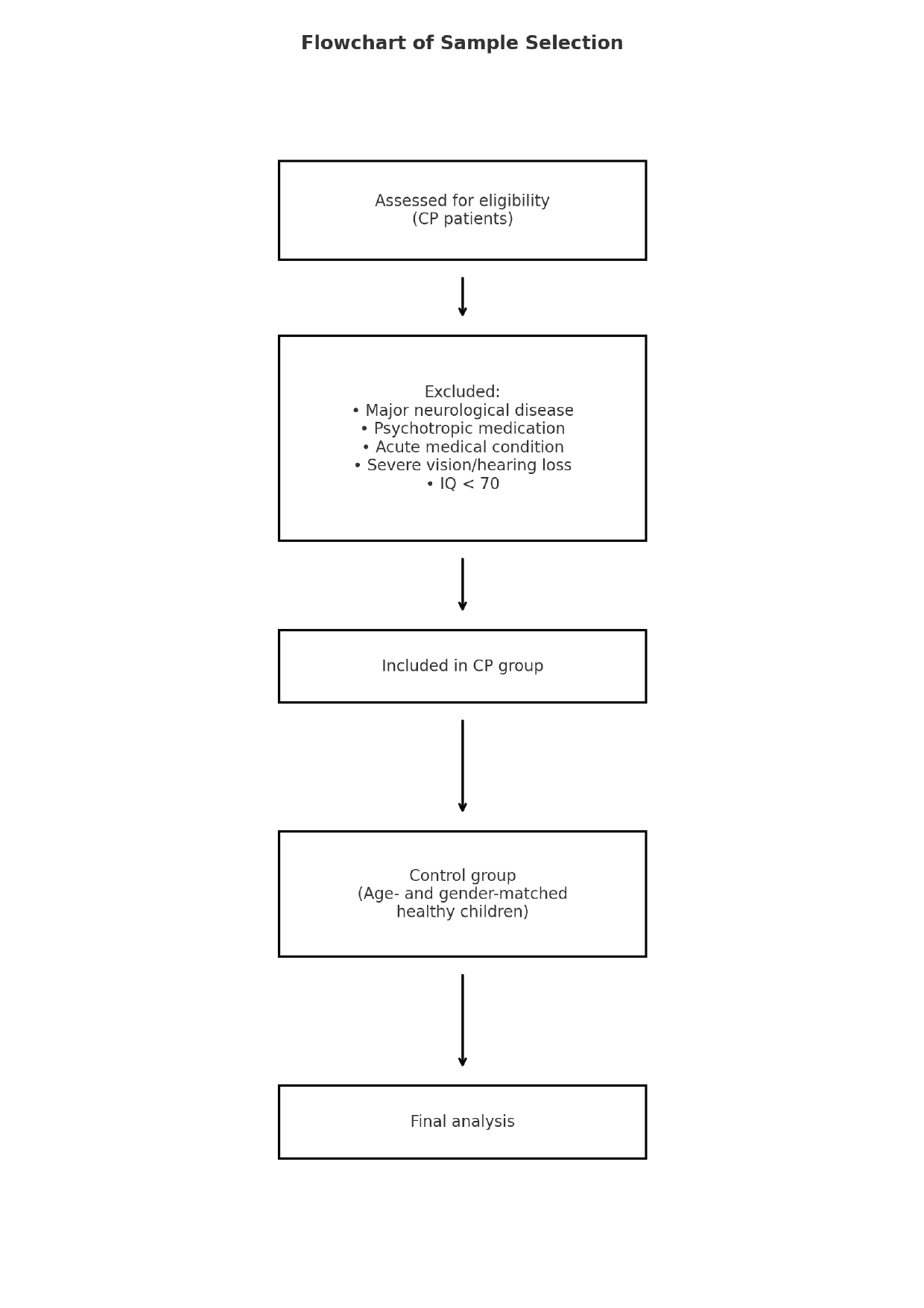

Supplement: Supplementary Figure S1 — Flowchart illustrating the recruitment, screening, exclusion, and final inclusion of participants in the study. CP = Cerebral Palsy. [file Image1.jpeg]
